# Transcranial versus direct electrical stimulation for intraoperative motor-evoked potential monitoring: Prognostic value comparison in asleep brain tumor surgery

**DOI:** 10.3389/fonc.2022.963669

**Published:** 2022-09-29

**Authors:** Luca Viganò, Vincenzo Callipo, Marta Lamperti, Marco Rossi, Marco Conti Nibali, Tommaso Sciortino, Lorenzo Gay, Guglielmo Puglisi, Antonella Leonetti, Gabriella Cerri, Lorenzo Bello

**Affiliations:** ^1^ Neurosurgical Oncology Unit, Department of Oncology and Hemato-Oncology, Università degli Studi di Milano, IRCCS Galeazzi-Sant'Ambrogio, Milano, Italy; ^2^ Motor, Cognition and Action Laboratory, Department of Medical Biotechnology and Translational Medicine, Università degli Studi di Milano, Milano, Italy

**Keywords:** brain tumor, intraoperative monitoring (IOM), motor evoked potential (MEP), transcranial electrical stimulation (TES), direct cortical stimulation (DCS), corticospinal tract (CST)

## Abstract

**Objective:**

Safe resection of gliomas involving motor pathways in asleep-anesthesia requires the combination of brain mapping, to identify and spare essential motor sites, and continuous monitoring of motor-evoked potentials (MEPs), to detect possible vascular damage to the corticospinal tract (CST). MEP monitoring, according to intraoperative neurophysiology societies, is generally recommended by transcranial electrodes (TES), and no clear indications of direct cortical stimulation (DCS) or the preferential use of one of the two techniques based on the clinical context is available. The main aim of the study was to identify the best technique(s) based on different clinical conditions, evaluating the efficacy and prognostic value of both methodologies.

**Methods:**

A retrospective series of patients with tumors involving the motor pathways who underwent surgical resection with the aid of brain mapping and combined MEP monitoring *via* TES and DCS was evaluated. Irreversible MEP amplitude reduction (>50% compared to baseline) was used as an intraoperative warning and correlated to the postoperative motor outcome. Selectivity, specificity, positive predictive value (PPV), and negative predictive value (NPV) were computed for both techniques.

**Results:**

Four hundred sixty-two patients were retrospectively analyzed, and only 1.9% showed a long-term motor impairment. Both TES and DCS obtained high specificity and NPV for the acute and 1-month motor deficit. Sensitivity was rather low for the acute deficit but excellent considering the 1-month follow-up for both techniques. DCS was extremely reliable in predicting a postoperative motor decline (PPV of 100% and 90% for acute and long-term deficit, respectively). Conversely, TES produced a high number of false-positive results, especially for long-term deficits (65, 87.8% of all warnings) therefore obtaining poor PPV values (18% and 12% for acute and 1-month deficits, respectively). TES false-positive results were significantly associated with parietal tumors and lateral patient positioning.

**Conclusions:**

Data support the use of mapping and combined monitoring *via* TES and DCS. The sole TES monitoring is reliable in most procedures but not in parietal tumors or those requiring lateral positioning. Although no indications are available in international guidelines, DCS should be recommended, particularly for cases approached by a lateral position.

## 1 Introduction

The aim of modern neurosurgical oncology is maximal safe tumor resection (1, 2). For tumors involving motor areas, the preservation of descending motor pathways is of utmost importance because motor deficits dramatically impact patients’ quality of life, have a poor expectancy of recovery, and decrease patients’ eligibility for adjuvant therapies (3–6). A safe resection requires combining brain mapping and monitoring techniques (7, 8). Brain mapping, by means of direct electrical stimulation (DES), reliably identifies essential cortical and subcortical motor sites belonging to the corticospinal tract (CST) (9–11). Mapping alone, however, does not detect vascular damage, such as ischemic events following injuries to deep small penetrating end arteries (12, 13), and thus is not sufficient to preserve motor function. Mapping is therefore paired with a continuous real-time intraoperative assessment of CST integrity by monitoring motor-evoked potentials (MEPs), whose alterations parallel changes in vascular supply to CST territories. According to the intraoperative neurophysiology society recommendations for surgeries performed asleep, MEP monitoring is generally implemented with transcranial electrodes (TES): scalp electrodes for TES are derived from the international 10/20 system (C1, C2, C3, C4, and Cz) and recording electrodes placed in at least three muscles in the upper and lower limb to detect MEPs (14). Alternatively, direct cortical stimulation (DCS) delivering short train-of-five pulses, To5 (15), is delivered by subdural stimulating grids directly placed over the convexity of the primary motor cortex (M1). With both techniques, MEP amplitude reduction (>50% compared to baseline) represents the main predictor of transient or permanent motor deficits (16) and, at the first warning sign, should be reported to the surgeon to undertake the proper measure to reduce the possible risk. Across neurosurgical teams, the choice between TES/DCS or their combined use is strictly dependent on the advantages and pitfalls of the two techniques, the experience or preferences of the team neuromonitoring, the clinical conditions, and costs. TES allows continuous monitoring of both hemispheres from the beginning to the end of surgery; DCS constrains the monitoring only to the ipsilesional M1 from the dura opening to closure. The efficacy of TES electric field over M1 is negatively affected by brain shift and cerebrospinal fluid (CSF) decrease (17); moreover, TES suffers from poor stimulation focality and, to obtain reliable MEPs, requires high current intensities. These factors may cause false warnings, i.e., MEP alteration in the absence of actual CST damage or, conversely, false-negative results: due to the high intensity required, the spread of current in deep white matter may indeed stimulate axons downstream of the vascular damage, resulting in unchanged MEPs despite a CTS lesion. Despite the fact that DCS grids may shift slightly during the procedure, DCS allows for focal stimulation at a lower current intensity, which reduces the occurrence of the above-mentioned bias.

At present, although several reports have explored the TES and DCS prognostic value (18), most guidelines recommend the use of TES for monitoring, and no clear indications are reported on DCS nor on the preferential use of one of the two techniques based on the clinical context.

In recent years, we have routinely performed intraoperative brain monitoring combining TES and DCS for supratentorial tumors. We here present a retrospective analysis of about 500 patients admitted between 2018 and 2020 evaluating the selectivity, specificity, positive predictive value (PPV), and negative predictive value (NPV) of TES and DCS, directly compared within the same sample of patients. Irreversible MEP amplitude decrease (>50% compared to baseline) was recorded with both techniques and selected as an intraoperative warning sign of possible CST damage (14, 16, 19). This parameter was correlated with the postoperative motor outcome to evaluate the prognostic value of TES and DCS in predicting the onset of a motor deficit. This analysis provided useful information to identify the best choice, between the two technique(s), to be applied in different clinical conditions. In addition, the efficacy of TES and DCS was discussed considering technical issues and clinical variables.

## 2 Materials and methods

### 2.1 Patients

A total of 500 patients undergoing supratentorial resection for a tumor involving motor pathways using a combined TES/DCS MEP monitoring between 2018 and 2020 were screened for inclusion. Exclusion criteria were as follows: severe preoperative motor deficit (MRC scale <4), as altered motor excitability may be a confounding factor in MEP value interpretation in these procedures; suboptimal subdural grid positioning (as for resection of tumors within the M1 hand-knob); and postoperative SMA syndrome, as the occurrence of the motor deficit is unrelated to DCS MEP loss (20). Only procedures with optimal TES and DCS electrode placement before corticectomy were included. Clinical, imaging, and histomolecular features of patients and tumors were collected. Patients gave formal consent to the procedures and the study (IRB-1299).

### 2.2 MR acquisition

Axial-three-dimensional-(3D)-FLAIR, post-Gd-three-dimensional-T1-weighted, and DWI-ADC diffusion-weighted images were collected preoperatively on a Siemens Magnetom Verio 3.0-T system. Patients underwent postoperative volumetric-FLAIR and post-GdT1-weighted imaging both within 48 h and 2 months after surgery for extent-of-resection (EOR) estimation (1). An immediate postoperative DWI was acquired to evaluate ischemia (21).

### 2.3 Surgical procedure and brain mapping

All surgeries were performed with the aid of brain mapping and monitoring, and resection was stopped according to functional boundaries. Based on tumor localization, patients were positioned supine (frontal, precentral, temporal, and frontotemporal tumors) or lateral (parietal and temporoparietal tumors). The craniotomy exposed the tumor area and a limited portion of the surrounding cortex, always looking either for the site(s) evoking the lowest cortical motor threshold for identifying the most efficient position of the subdural grid *via* direct high-frequency DES (HF-DES) mapping or grid electrode stimulation. Also, in the case of temporal or parietal tumors, a subdural grid placement was performed. Neuronavigation was available for surgical planning and intraoperative use. An asleep or asleep-awake-asleep anesthesia was applied according to clinical needs (8, 22, 23). At the beginning of surgery, for cortical motor mapping, HF-DES was applied, when needed and feasible, at a cortical level to identify M1, where a grid electrode was placed for constant MEP monitoring. HF-DES was delivered using a constant current monopolar stimulator (straight tip, 1.5 mm diameter, Inomed, with reference/ground on the skull overlying the central sulcus) in trains of 5 (To5) constant anodal current pulses (pulse duration: 5 ms, interstimulus interval ISI: 3–4 ms). For asleep procedures, HF-DES motor mapping was used to identify the safe entry zone to start the corticectomy and was also applied subcortically (cathodal stimulation) to identify M1 fibers until a threshold of 3 mA was reached (9–11, 24, 25). For awake procedures, in addition to HF-DES motor mapping, low-frequency DES (LF-DES) was applied either cortically or subcortically to identify and preserve sites in which DES evoked praxis, language, visual, and cognitive interferences (26–29). LF-DES was delivered by a bipolar probe with a 5-mm distance tip (60 Hz, pulse width = 0.5 ms, biphasic current, 1–4 s of stimulation). The lowest current intensity that interfered with language, cognitive, or praxis tasks over the precentral gyrus was applied throughout the cortical and subcortical mapping. In the awake cases, sites evoking language, cognitive, praxis, and visual responses (requiring patient cooperation) were identified at the beginning of the resection, obtaining a partial functional disconnection of the tumor mass (duration of the awake phase: 20–30 min on the average). After the patients were again put under general anesthesia, and sites evoking M1 fiber motor responses were then identified during the asleep phase of resection, as in the completely asleep cases, coupling motor mapping with HF-DES MEP monitoring.

### 2.4 Neurophysiological monitoring

The integrity of the descending motor pathways was monitored throughout the procedure by using a To5 monitoring technique (pulse duration, 0.5–0.8 ms; ISI, 2–4 ms; repetition rate, 1–1.5 Hz) delivered to M1 to elicit MEPs, either by TES and DCS. TES was delivered through corkscrew-like subcutaneous electrodes placed, according to surgical flap, at C2–C1 or C3–C4 and Cz (10/20 system). DCS was delivered through a 4/6-contact subdural strip electrode placed over M1, localized with HF-DES and SSEP phase reversal. Motor threshold (MT) was established after dura opening as the lowest current intensity evoking reproducible MEP (peak-to-peak amplitude, >50 μV) for both TES and DCS. For DCS, monitoring was performed with the electrode showing the lowest MT. MEPs were recorded by pairs of subdermal hook needle electrodes (Technomed) inserted into the following muscles: bilateral orbicularis oris, bilateral hemitongue, mentalis, biceps brachii, flexor carpi radialis, extensor digitorum communis, abductor digiti minimi, first dorsal interosseous, bilateral abductor pollicis brevis, quadriceps, bilateral tibialis anterior, and flexor hallucis brevis. All muscles were connected to a multichannel EMG recording (2,000 Hz sample frequency, ISIS-IOM, InomedGmbH) (7). MEP amplitude reduction (>50% compared to baseline) was used as a significant warning sign, immediately reported to the surgeon, and finally recorded. TES warnings were considered reliable only when the decrease of MEP amplitude at MT parameters was registered in the affected hemisphere and paired with unchanged MEP amplitude in the contralesional hemisphere.

ECoG and free-running EMG allowed for the detection and avoidance of intraoperative seizures: at the first ictal sign, stimulation was stopped and cold irrigation was applied over the cortex for seizure abortion. Whenever the seizures spread to the whole hemibody, a bolus infusion of propofol (4 ml on average) was delivered.

In all patients, monitoring included simultaneous acquisition of continuous EEG, ECoG, free-running EMG, MEPs, and somatosensory-evoked potentials (SEPs). EEG (C1, C2, C3, C4, and Cz) and ECoG (4/6-contact subdural grid over the precentral gyrus) were recorded to detect seizures, after discharges during stimulation, depth of anesthesia, and thus to titrate the level of anesthetics to maintain optimal cortical excitability.

### 2.5 Data analysis

Demographic and clinical features at admission included age, sex, symptoms, and previous treatments. Tumor histological variables include histology and molecular profile (IDH status, codeletion, ATRX mutation). Motor function was assessed preoperatively, on the 5th day (immediate) and 1 month (permanent) after surgery using the MRC scale. The postoperative decline of motor function was considered mild (MRC reduction ≤1) or severe (MRC reduction >1).

EOR was calculated on postoperative MRI (within 48 hours), targeting postcontrast MRI for enhancing lesions or FLAIR for nonenhancing lesions and classified based on residual tumor volume (RTV) as total (RTV = 0), subtotal (0 < RTV ≤ 5 cm^3^), and partial (RTV > 5 cm^3^). Supratotal resection was defined as resection extending outside the tumor border as seen in the preoperative MR (FLAIR for nonenhancing lesions, postcontrast T1 for enhancing ones).

Immediate postoperative diffusion-weighted MRI scans were also performed to detect ischemia, and the number of DWI abnormalities was categorized as previously reported (21).

All >50% decrements of MEP amplitude recorded during the procedure were stored. Only irreversible MEP amplitude reduction detected during the resection and persisting beyond the end of resection and hemostasis (>50% compared to the amplitude recorded after dura opening and before corticectomy) was considered a positive TES/DCS sign, used for analysis and compared with postoperative motor function outcome. Irreversible MEP amplitude decrements were categorized in MEP reduction (amplitude drop from >50% to <90%) and MEP loss (drop >90%). Sensitivity, specificity, PPV, and NPV were computed for both TES and DCS at 5 days and 1 month. True negative (TN; negative TES/DCS result in the absence of postoperative motor deterioration), true positive (TP; positive TES/DCS result and occurrence of postoperative motor deterioration), false negative (FN; negative TES/DCS result and occurrence of postoperative motor deterioration), and false positive (FP; positive TES/DCS result in absence of postoperative motor deterioration) were computed. Considering the high occurrence of false TES warnings in the analyzed sample, TES TP and FP results at 1 month were correlated with clinical variables, leading to brain shift and CSF decrease, i.e., the factors possibly affecting TES reliability according to extensive neurosurgical experience and preliminary reports (17, 30). The variables selected were tumor localization, patient positioning (supine vs. lateral), and tumor volume, the latter impacting on time of surgery and volume of resected tissue. The volume of each brain lesion of TP and FP TES was manually delineated on the preoperative MR image using ITK-SNAP (FLAIR for nonenhancing lesion, postcontrast T1 for enhancing ones), and both were registered to MNI by means of lesion masking approach using the Clinical Toolbox in SPM (enantiomorphic normalization) (31). Overlap maps of the two groups were computed. To investigate if a tumor location may predict the occurrence of false positive results, nonparametric statistics were performed on lesion segmentations using FSLs randomized with 5,000 permutations and threshold-free cluster enhancement (TFCE) to correct for multiple comparisons (32). One-sample *t*-tests were used with variance smoothing to assess which tumor localization was associated with the occurrence of TES FP. The family-wise error threshold was set at *p* < 0.05.

### 2.6 Statistical analysis

For categorical data, Fisher’s exact (two-category) tests were used. ANOVA was used for comparisons between continuous variables. Analysis was performed with IBM SPSS software (IBM Corp., Armonk, NY).

## 3 Results

### 3.1 Patients

In the study period, 500 patients with a tumor involving motor pathways were treated with the aid of motor mapping and monitoring combining TES and DCS. Twelve patients with a severe preoperative motor deficit (MRC <4), 15 patients with postoperative SMA syndrome and 11 patients with tumors infiltrating the M1 hand-knob region were excluded from the analysis. In total, 462 patients (mean age 51; SD 14.7; 262 had a tumor in the left hemisphere; 221 were women) fulfilled the inclusion criteria, had full clinical and imaging data available, and were used for the analysis. Their clinical and imaging features are reported in **Table 1**.

**Table 1:** Demographic and clinical information.

Variable	Value	%
Sex		
Male	241	52
Female	221	48
Age (years)		
Mean	51	
Range	18–89	
Side		
Left	262	57
Right	200	43
Location		
Frontal	155	33.5
Temporal	94	20.4
Insular	79	17.1
Parietal	134	29
Histology		
LGG	189	41
HGG	170	37
Meningioma	54	12
Metastasis	39	8
Other^*^	10	2
Previous treatment		
Yes	156	44
No	306	66
Type of anesthesia		
Asleep	273	59
Awake	189	41
EOR		
Supratotal	95	20.6
Total	339	73.4
Subtotal	28	6
Partial	0	0

^*^Cavernous angioma (five, 1%), cysts (three, 0.6%), and DNET (two, 0.4%). Previous treatment (surgery).

### 3.2 Intraoperative monitoring results and motor outcome

Both TES and DCS MEP monitoring were successfully recorded in all patients. Monitoring was continuously performed at threshold parameters (MT values for TES: mean 60 mA, SD 12.3; MT values for DCS: mean 7 mA, SD: 3.1), and no analyzed MEP variations were related to altered cortical excitability or electrode mispositioning.

Out of 462 patients, a >50% MEP reduction was recorded in 121 (26.2%) patients during tumor resection. They were immediately reported to the surgeon who stopped resection and took all the measures to allow full MEP restoration. The reduction was reversible in 47 procedures (10.1%) (10 detected by both techniques, 11 by only TES, and 26 by only DCS). An irreversible MEP amplitude reduction was instead recorded during 74 procedures with TES (16%) and only in 10 patients with DCS (2.2%) (**Table 2**). Within the irreversible warnings detected by TES, an amplitude reduction (<90%) was found in 27 patients (36.5%), while a MEP loss (>90%) was found in 47 patients (63.5%). Irreversible DCS MEP reduction (<90%) occurred in two procedures (20%), while a MEP loss (>90%) was recorded in the remaining eight patients (80%). All complete MEP losses, either with TES or DCS, were recorded for both lower- and upper-limb muscles.

**Table 2:** Postoperative motor outcome and intraoperative findings.

Variable	Value	%
MEP reversible reduction (TES)		
Yes	21	4.5
No	441	95.4
MEP reversible reduction (DCS)		
Yes	36	7.8
No	426	92.2
MEP irreversible reduction (TES)		
Yes	74	16
No	388	84
MEP irreversible reduction (DCS)		
Yes	10	2.2
No	452	97.8
Preop MRC score		
5	437	94.5
4	25	4.5
MRC score reduction at 5 days		
Mild (reduction ≤1)	19	4
Severe (reduction >1)	11	2.4
MRC score reduction at 1 month		
Mild (reduction ≤1)	2	0.4
Severe (reduction >1)	7	1.5

In the case of TES, the significant MEP decrement was preceded by MEP fluctuations in 32 cases. In the case of DCS, it occurred within a few seconds (3–5) in nine out of 10 cases and was preceded by fluctuations in one patient only. Notably, a significant decrement recorded by DCS was always paralleled and confirmed by TES, while the opposite was not always verified: a significant decrement detected by TES was indeed associated with a DCS decrement in 10 cases only. All irreversible warnings, irrespectively by TES or DES, were recorded during the resection phase and mostly subcortically while the last deep portion of the tumor was removed, close to CST fibers or in the deep insula. No irreversible MEP warnings were recorded after dura closure (i.e., after removal of DES strip), by TES only.

The postoperative analysis of patients included in the study showed an immediate decline in postoperative MRC score only in 30 patients (6.5%) and specifically mild (MRC reduction ≤1) in 19 (63.3%) and severe (MRC reduction >1) in 11 (36.7%). Out of 30 patients with postoperative MRC reduction, 21 (70%) showed full recovery of motor abilities at 1-month follow-up. In the whole sample, a mild motor decline persisted for 1 month in two patients (0.4%), and only seven patients (1.5%) suffered a long-term severe motor deficit (**Table 2**).

#### 3.2.1 Prediction of acute motor decline

**Figure 1:**
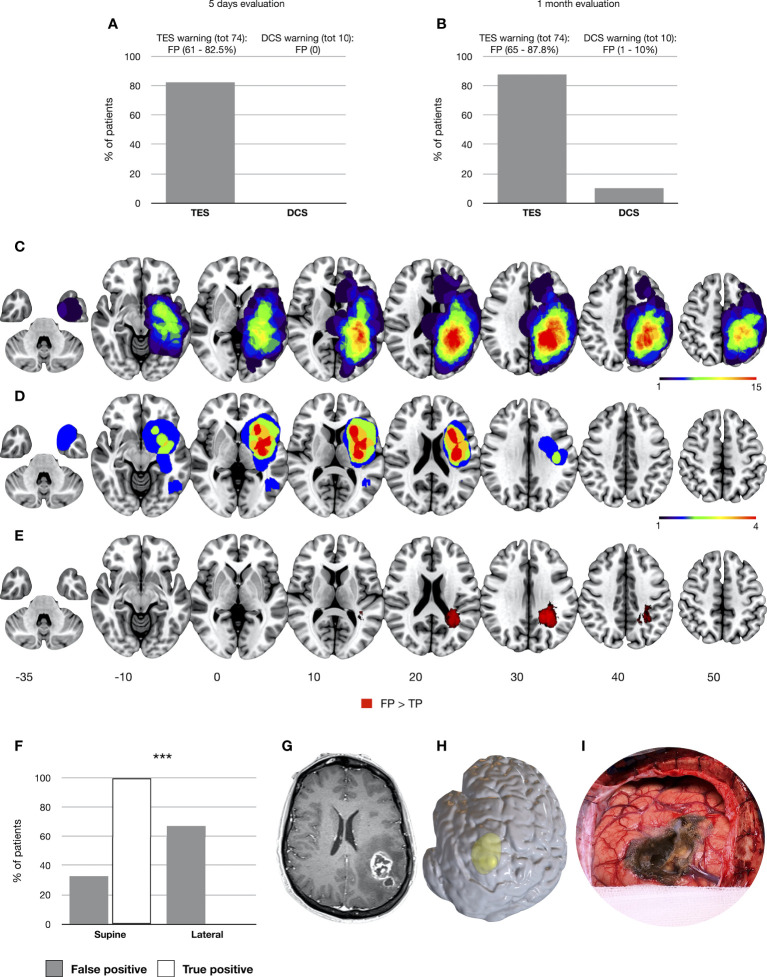
Occurrence of false-positive results for TES and DCS considering the 5-day **(A)** and 1-month follow-up **(B)**. Tumor volumes of patients with TES FP and TP results (1-month follow-up) are overlapped respectively in **(C, D)**. The significant cluster predicting the occurrence of TES FP results is displayed in **(E)** (TFCE, *p* = 0.05). All tumor volumes, normalized to the MNI template, are visualized on left hemisphere axial slices. In **(F)** the distribution of supine and lateral craniotomies is reported between the FP and TP patients. Finally, in **(G)** (left panel, postcontrast T1-weighted images), a case of a high-grade glioma located in the left parietal lobe is presented in which a TES intraoperative MEP amplitude decreased but preserved postoperative motor status was recorded. In **(H)**, a 3D render of the preoperative T1 is presented to show the lateral head positioning adopted for the resection. **(I)** A picture of the intraoperative field showing the brain shift and the presence of an air layer over the M1 convexity. ^∗∗∗^p < .001.

Out of 74 intraoperative TES warnings, only 13 (17.5%) predicted an acute postoperative motor impairment (mild in five patients; severe in eight), so 61 (82.5%) were FP results (**Figure 1A**). No significant MEP warnings were recorded with TES in 388 patients: most of them (371, 95.6%) coherently showed preserved motor abilities immediately after surgery, while 17 patients (4.4%) showed motor deterioration (mild in 14 and severe in three) and to were thus considered FN.

Notably, MEP warnings detected with DCS correlated on the 5th postoperative day with an MRC score decline in all patients (10, 100%; two mild and eight severe MRC reduction). No FP results were recorded. In 452 patients, no DCS irreversible MEP amplitude reduction occurred. Within them, in 20 patients (4.4%), a postoperative motor decline occurred (mild in 17 and severe in three), to be thus considered FN. Conclusively, the efficacy of TES and DCS to predict the onset of acute motor deficits was rather low, with sensitivity values of 43% and 33%, respectively. However, as distinguishing features between the two techniques, when an irreversible MEP amplitude drop was recorded, the probability of predicting a postoperative motor deficit was higher for DCS (PPV 100%) compared to TES (PPV 18%). Both techniques showed high specificity (TES 86%; DCS 100%) and NPV (96% for both).

#### 3.2.2 Prediction of 1-month motor decline and factors associated with TES false-positive results

The motor assessment at the 1-month follow-up revealed a worst ratio between TES TP (nine, 12.2%—two mild and seven severe MRC reduction) and FP (65, 87.8%) (**Figure 1B**). Therefore, TES PPV for the 1-month motor deficit was extremely low (12%). Overlap maps showed a different lesion distribution between the TP group, mainly involving the insular region, and the FP group, with the highest overlap in the superior and inferior parietal lobe (**Figures 1C, D**). Regression on lesion segmentations showed a clear association with a cluster of voxels (*n* = 16,092, TFCE, p-fcwer <0.05) located in the deep white matter of the inferior parietal lobe and the FP TES group (**Figure 1E**). All TP procedures were characterized by supine positioning, and the FP group was significantly associated with lateral head positioning (*p* = 0.00001) (**Figure 1F**). **Figures 1G–I** shows a representative patient with a left high-grade glioma operated with a lateral head positioning for which a TES FP result was recorded. The tumor volume was not different between TP (median 28.66 cm^3^; range, 2.85–115.95 cm^3^) and FP (median 34.3 cm^3^; range, 6.92–200.8 cm^3^).

All 388 patients with negative TES results preserved motor abilities at 1 month. Compared to acute motor assessment, TES sensitivity for long-term motor deficits was significantly higher (100%), and specificity and NPV confirmed high performances (86% and 100%).

At the 1-month follow-up, MEP warnings detected with DCS correlated with a postoperative MRC score decline in nine out of 10 patients. With respect to the immediate postoperative phase, one patient with a parietal tumor infiltrating Berger zone 1 (21) with lower-limb hyposthenia fully recovered. The remaining eight patients showed postoperative DWI alteration. Compared to the poor predictive power of TES, DCS showed a PPV of 90%. Out of 453 patients with negative intraoperative DCS results, no FN was found. DCS sensitivity, specificity, and NPV for long-term motor deficit were 100% all. **Table 3** displays all TES/DCS performance parameters.

**Table 3:** TES and DCS performance.

Variable	TES	DCS
TP		
5 days	13	10
1 month	9	9
FP		
5 days	61	0
1 month	65	1
TN		
5 days	371	432
1 month	388	452
FN		
5 days	17	20
1 month	0	0
Sensitivity		
5 days	43%	33%
1 month	100%	100%
Specificity		
5 days	86%	100%
1 month	86%	100%
PPV		
5 days	18%	100%
1 month	12%	90%
NPV		
5 days	96%	96%
1 month	100%	100%

TP, true positive; FP, false positive; TN, true negative; FN, false negative; PPV, positive predictive value; NPV, negative predictive value.

## 4 Discussion

Resection of tumors involving motor pathways in asleep anesthesia requires the combined use of motor mapping and monitoring to perform an efficient and safe resection. The recommended standard for monitoring is TES-MEP delivered by skull corkscrew electrodes. The modality of electrode placement, stimulation parameters, recording electrodes, measurement, and alarm criteria have been reported by international guidelines (14). Despite the body of data available on DCS monitoring usage (19, 20, 33–36), this technique is still not included as a standard tool in most guidelines. No clear indications are available on the preferential use of one of the two techniques based on the clinical context.

To our knowledge, this study represents the report with the largest cohort of brain tumor patients in which TES and DCS monitoring were combined in all procedures, allowing a direct comparison of their sensitivity, specificity, PPV, and NPV within the same patients. To this aim, irreversible MEP amplitude decrease (>50% compared to baseline) was correlated with the acute and 1-month postoperative motor outcome. The main aim was to identify the best technique(s) match for the different sets of clinical conditions.

The diagnostic accuracy of both techniques, although never systematically investigated and being strictly dependent on inclusion criteria selection (e.g., lesion location, combined mapping strategy, and different warning criteria adopted), was reported as similar between both techniques (18). Consistent with previous reports assuming a predictive criterion of >50% MEP amplitude reduction (12, 37–41), we found TES/DCS high specificity and NPV for both the acute and 1-month motor deficit. For the latter, no false-negative results were found. This means that, regardless of the technique used, the absence of relevant intraoperative warning reliably indicates that the CST is still functioning. Results also showed a trend of increasing sensitivity from the immediate postoperative phase and 1-month follow-up, reaching 100% for both methods. These data reflect that only 1.9% of the whole sample of patients suffered persistent postoperative motor impairment, and most patients showing a postoperative MRC score decline fully recovered within a few weeks (21 out of 30, 70%). According to our results, TES and DCS significantly diverged in relation to PPV, i.e., the ability to predict, given an intraoperative irreversible MEP amplitude reduction, a postoperative deficit in motor performance. If DCS appeared extremely reliable (100% and 90% of PPV respectively at 5 days and 1-month follow-up), TES monitoring resulted in a high number of false-positive results, especially for long-term deficits (65, 87.8% of all warnings), therefore showing poor PPV values (18% and 12% respectively at 5 days and 1-month follow-up).

The analysis of TES positive (false positive over true positive) overlap maps reveals a clear dissociation between the tumor location of FP (mainly extending in the superior and inferior parietal lobe) and TP (mainly overlapping within the insula). Regression on lesion segmentations confirmed a cluster of voxels in the deep white matter of the inferior parietal lobe as a significant predictor of false-positive TES warnings. Notably, lateral head positioning (adopted for parietal resections) was associated with the FP group. We suggest this finding be strictly correlated with a possible reduction of electrical field effectiveness over M1 in parietal craniotomies requiring a lateral positioning. CSF decrease and brain shift, occurring after dura opening and increasing during the progression of the resection, cause the formation of an air layer above the M1 convexity, compromising transcranial conductivity. Data on TES electrical field visualization by means of the finite element method confirm the suboptimal ignition on M1 for parietal craniotomies and directly support our clinical results (17, 30).

In our series, during parietal craniotomies, DCS was continuously paired with TES and allowed the online detection of TES false warnings. Should only TES have been available during these procedures, resection would have been interrupted prematurely, possibly leading to a poorer extent of resection and, therefore, to a worst oncological outcome (1).

Only nine patients showed persistent motor deficit at 1 month (1.9%), which were all predicted both by both TES and DCS. Within this group (TP), all patients had insular tumors and documented DWI alterations along motor pathways. During these procedures, MEP changes reliably anticipated the onset of motor decline during dissection along middle cerebral, lenticulostriate, and/or anterior choroidal artery perforators; however, in most of these cases, the warning occurred abruptly (in less than 3 s), a tight time window preventing the identification of a hierarchic series of predictive changes (13, 19). However, the availability of such information enabled all measures (saline irrigation, arterial pressure increase) to be taken promptly to reduce the functional impact of ischemic events.

When considering the limitations of this study, we must first point out that this is a retrospective study and is limited by selection bias. Second, the assessment of motor outcome performance relies on the MRC score only, while other more refined assessments may reveal deficits neglected by this assessment. As the main aim of the study was to evaluate the sensitivity and specificity of TES and DCS by correlating the intraoperative warnings (irreversible MEP decrement or loss) with the postoperative motor deficit, only procedures with an optimal TES and DCS electrode placement (performed before corticectomy) and with stable MEP identification were included. The feasibility and safety of the two techniques based on clinical conditions go beyond the scope of the study and are reported by previous data (16, 33, 34). Among the reported alarm criteria (18), only the >50% MEP reduction was chosen as the cutoff value for the analysis. While the use of TES requires the placement of corkscrews with limited cost, the use of DCS requires the correct placement of a cortical strip, which increases the global cost of surgery.

Globally considered, this study showed a higher performance of DCS in predicting postoperative motor impairment (PPV) compared to TES. Overall, TES showed high reliability but compromised efficacy in parietal resection requiring a lateral positioning, correlating with the occurrence of a high number of false-positive results. Conversely, DCS is reliable in all locations; despite being associated with a higher cost, it should be recommended, particularly for resection of parietal tumors or for cases approached by a lateral position.

## Data availability statement

The raw data supporting the conclusions of this article will be made available by the authors, without undue reservation.

## Ethics statement

The studies involving human participants were reviewed and approved by IRB-1299. The patients/participants provided their written informed consent to participate in this study.

## Author contributions

Conceptualization: LB, LV, GC, VC, and ML. Methodology: LB and LV. Formal analysis: LV, GP, and AL. Investigation: LB, LV, VC, ML, MR, MC, TS, and LG. Data curation: VC, ML, GP, and AL. Writing – Original Draft: LV. Writing – Review and Editing: LB, GC, VC, and ML. Visualization: LV. Supervision: LB and GC. Project administration: LB and GC. Funding acquisition: LB. All authors contributed to the article and approved the submitted version.

## Funding

This work was supported by the AIRC grant (No. G18482) awarded to LB.

## Conflict of interest

The authors declare that the research was conducted in the absence of any commercial or financial relationships that could be construed as a potential conflict of interest.

## Publisher’s note

All claims expressed in this article are solely those of the authors and do not necessarily represent those of their affiliated organizations, or those of the publisher, the editors and the reviewers. Any product that may be evaluated in this article, or claim that may be made by its manufacturer, is not guaranteed or endorsed by the publisher.

## Abbreviations

CSF, cerebrospinal fluid; CST, corticospinal tract; DCS, direct cortical stimulation; DES, direct electrical stimulation; ECoG, electrocorticography; EOR, the extent of the resection; FN, false negative; FP, false positive; HF-DES, high-frequency direct electrical stimulation; IOM, intraoperative monitoring; LF-DES, low-frequency direct electrical stimulation; M1, primary motor area; MEP, motor evoked potential; MRC, Medical Research Council; MT, motor threshold; NPV, negative predictive value; PPV, positive predictive value; RTV, residual tumor volume; SEP, somatosensory evoked potential; TES, transcranial electrical stimulation; TFCE, threshold-free cluster enhancement; TN, true negative; TP, true positive.

## References

[1] Rossi M Gay L Ambrogi F Conti Nibali M Sciortino T Puglisi G. Association of supratotal resection with progression-free survival, malignant transformation, and overall survival in lower-grade gliomas. Neuro Oncol (2021) 23(5):812–26. doi: 10.1093/neuonc/noaa225 PMC8099476 33049063

[2] Hervey-Jumper SL Berger MS. Evidence for improving outcome through extent of resection. Neurosurg Clin N Am (2019) 30(1):85–93. doi: 10.1016/j.nec.2018.08.005 30470408

[3] Ius T Angelini E Thiebaut de Schotten M Mandonnet E Duffau H. Evidence for potentials and limitations of brain plasticity using an atlas of functional resectability of WHO grade II gliomas: Towards a “minimal common brain”. NeuroImage (2011) 56(3):992–1000. doi: 10.1016/j.neuroimage.2011.03.022 21414413

[4] Weller M van den Bent M Hopkins K Tonn JC Stupp R Falini A. EANO guideline for the diagnosis and treatment of anaplastic gliomas and glioblastoma. Lancet Oncol (2014) 15(9):e395–403. doi: 10.1016/S1470-2045(14)70011-7 25079102

[5] Weller M van den Bent M Preusser M Le Rhun E Tonn JC Minniti G. EANO guidelines on the diagnosis and treatment of diffuse gliomas of adulthood. Nat Rev Clin Oncol (2021) 18(3):170–86. doi: 10.1038/s41571-020-00447-z PMC7904519 33293629

[6] Leonetti A Puglisi G Rossi M Viganò L Conti Nibali M Gay L. Factors influencing mood disorders and health related quality of life in adults with glioma: A longitudinal study. Front Oncol (2021) 11:662039. doi: 10.3389/fonc.2021.662039 34094955 PMC8173148

[7] Bello L Riva M Fava E Ferpozzi V Castellano A Raneri F. Tailoring neurophysiological strategies with clinical context enhances resection and safety and expands indications in gliomas involving motor pathways. Neuro-Oncology (2014) 16(8):1110–28. doi: 10.1093/neuonc/not327 PMC4096171 24500420

[8] Rossi M Sciortino T Conti Nibali M Gay L Viganò L Puglisi G. Clinical pearls and methods for intraoperative motor mapping. Neurosurgery (2021) 88(3):457–67. doi: 10.1093/neuros/nyaa359 PMC7884143 33476393

[9] Raabe A Beck J Schucht P Seidel K. Continuous dynamic mapping of the corticospinal tract during surgery of motor eloquent brain tumors: evaluation of a new method: Clinical article. J Neurosurg (2014) 120(5):1015–24. doi: 10.3171/2014.1.JNS13909 24628613

[10] Rossi M Viganò L Puglisi G Conti Nibali M Leonetti A Gay L. Targeting primary motor cortex (M1) functional components in M1 gliomas enhances safe resection and reveals M1 plasticity potentials. Cancers (Basel) (2021) 13(15):3808. doi: 10.3390/cancers13153808 34359709 PMC8345096

[11] Rossi M Conti Nibali M Viganò L Puglisi G Howells H Gay L. Resection of tumors within the primary motor cortex using high-frequency stimulation: oncological and functional efficiency of this versatile approach based on clinical conditions. J Neurosurg (2019) 9:1–13. 10.3171/2019.5.JNS19453 31398706

[12] Neuloh G Pechstein U Schramm J. Motor tract monitoring during insular glioma surgery. J Neurosurg (2007) 106(4):582–92. doi: 10.3171/jns.2007.106.4.582 17432707

[13] Rossi M Gay L Conti Nibali M Sciortino T Ambrogi F Leonetti A. Challenging giant insular gliomas with brain mapping: Evaluation of neurosurgical, neurological, neuropsychological, and quality of life results in a Large mono-institutional series. Front Oncol (2021) 11:823. doi: 10.3389/fonc.2021.629166 PMC8019925 33828981

[14] Legatt AD Emerson RG Epstein CM MacDonald DB Deletis V Bravo RJ. ACNS guideline: Transcranial electrical stimulation motor evoked potential monitoring. J Clin Neurophysiol (2016) 33(1):42–50. doi: 10.1097/WNP.0000000000000253 26756258

[15] Taniguchi M Cedzich C Schramm J. Modification of cortical stimulation for motor evoked potentials under general anesthesia: technical description. Neurosurgery (1993) 32(2):219–26. doi: 10.1227/00006123-199302000-00011 8437660

[16] MacDonald DB. Overview on criteria for MEP monitoring. J Clin Neurophysiology (2017) 34(1):4–11. doi: 10.1097/WNP.0000000000000302 28045852

[17] Tomio R Akiyama T Toda M Ohira T Yoshida K. The impact of several craniotomies on transcranial motor evoked potential monitoring during neurosurgery. J Neurosurg (2017) 127(3):543–52. doi: 10.3171/2016.7.JNS152759 27715440

[18] Asimakidou E Abut PA Raabe A Seidel K. Motor evoked potential warning criteria in supratentorial surgery: A scoping review. Cancers (2021) 13(11):2803. doi: 10.3390/cancers13112803 34199853 PMC8200078

[19] Seidel K Beck J Stieglitz L Schucht P Raabe A. The warning-sign hierarchy between quantitative subcortical motor mapping and continuous motor evoked potential monitoring during resection of supratentorial brain tumors. J Neurosurg (2013) 118(2):287–96. doi: 10.3171/2012.10.JNS12895 23198802

[20] Giampiccolo D Parisi C Meneghelli P Tramontano V Basaldella F Pasetto M. Long-term motor deficit in brain tumour surgery with preserved intra-operative motor-evoked potentials. Brain Commun (2021) 3(1):fcaa226. doi: 10.1093/braincomms/fcaa226 33615216 PMC7884605

[21] Magill ST Han SJ Li J Berger MS. Resection of primary motor cortex tumors: feasibility and surgical outcomes. J Neurosurg (2017) 129(4):961–72. doi: 10.1093/neuonc/nox036.424 29219753

[22] Rossi M Puglisi G Nibali MC Viganò L Sciortino T Gay L. Asleep or awake motor mapping for resection of perirolandic glioma in the nondominant hemisphere? development and validation of a multimodal score to tailor the surgical strategy. J Neurosurg (2021) 136(1):16–29. 34144525 10.3171/2020.11.JNS202715

[23] Arzoine J Levé C Pérez-Hick A Goodden J Almairac F Aubrun S. Anesthesia management for low-grade glioma awake surgery: a European low-grade glioma network survey. Acta Neurochir (Wien) (2020) 162(7):1701–7. doi: 10.1007/s00701-020-04274-0 32128618

[24] Han SJ Morshed RA Troncon I Jordan KM Henry RG Hervey-Jumper SL. Subcortical stimulation mapping of descending motor pathways for perirolandic gliomas: assessment of morbidity and functional outcome in 702 cases. J Neurosurg (2018) 131(1):201–8. 10.3171/2018.3.JNS172494 30117770

[25] Schucht P Seidel K Jilch A Beck J Raabe A. A review of monopolar motor mapping and a comprehensive guide to continuous dynamic motor mapping for resection of motor eloquent brain tumors. Neurochirurgie (2017) 63(3):175–80. doi: 10.1016/j.neuchi.2017.01.007 28506487

[26] Rossi M Fornia L Puglisi G Leonetti A Zuccon G Fava E. Assessment of the praxis circuit in glioma surgery to reduce the incidence of postoperative and long-term apraxia: a new intraoperative test. J Neurosurg (2018) 130(1):17–27. doi: 10.3171/2017.7.JNS17357 29473778

[27] Viganò L Howells H Fornia L Rossi M Conti Nibali M Puglisi G. Negative motor responses to direct electrical stimulation: Behavioral assessment hides different effects on muscles. Cortex (2021) 137:194–204. doi: 10.1016/j.cortex.2021.01.005 33640851

[28] Conti Nibali M Leonetti A Puglisi G Rossi M Sciortino T Gay LG. Preserving visual functions during gliomas resection: Feasibility and efficacy of a novel intraoperative task for awake brain surgery. Front Oncol (2020) 10:1485. doi: 10.3389/fonc.2020.01485 32983985 PMC7492569

[29] Puglisi G Sciortino T Rossi M Leonetti A Fornia L Nibali MC. Preserving executive functions in nondominant frontal lobe glioma surgery: an intraoperative tool. J Neurosurgery (2018) 131(2):474–80. 10.3171/2018.4.JNS18393 30265193

[30] Tomio R Akiyama T Horikoshi T Ohira T Yoshida K. Visualization of the electric field evoked by transcranial electric stimulation during a craniotomy using the finite element method. J Neurosci Methods (2015) 256:157–67. doi: 10.1016/j.jneumeth.2015.09.014 26391774

[31] Nachev P Coulthard E Jäger HR Kennard C Husain M. Enantiomorphic normalization of focally lesioned brains. Neuroimage (2008) 39(3–3):1215–26. doi: 10.1016/j.neuroimage.2007.10.002 PMC2658465 18023365

[32] Winkler AM Ridgway GR Webster MA Smith SM Nichols TE. Permutation inference for the general linear model. Neuroimage (2014) 92:381–97. doi: 10.1016/j.neuroimage.2014.01.060 PMC4010955 24530839

[33] Krieg SM Schäffner M Shiban E Droese D Obermüller T Gempt J. Reliability of intraoperative neurophysiological monitoring using motor evoked potentials during resection of metastases in motor-eloquent brain regions: clinical article. J Neurosurg (2013) 118(6):1269–78. doi: 10.3171/2013.2.JNS121752 23521547

[34] Krieg SM Shiban E Droese D Gempt J Buchmann N Pape H. Predictive value and safety of intraoperative neurophysiological monitoring with motor evoked potentials in glioma surgery. Neurosurgery (2012) 70(5):1060–71. doi: 10.1227/NEU.0b013e31823f5ade 22067415

[35] Obermueller T Schaeffner M Shiban E Droese D Negwer C Meyer B. Intraoperative neuromonitoring for function-guided resection differs for supratentorial motor eloquent gliomas and metastases. BMC Neurol (2015) 15:211. doi: 10.1186/s12883-015-0476-0 26487091 PMC4618356

[36] Gempt J Krieg SM Hüttinger S Buchmann N Ryang YM Shiban E. Postoperative ischemic changes after glioma resection identified by diffusion-weighted magnetic resonance imaging and their association with intraoperative motor evoked potentials. J Neurosurg (2013) 119(4):829–36. doi: 10.3171/2013.5.JNS121981 23829818

[37] Boex C Haemmerli J Momjian S Schaller K. Prognostic values of motor evoked potentials in insular, precental, or postcentral resections. J Clin Neurophysiol (2016) 33(1):51–9. doi: 10.1097/WNP.0000000000000227 26840876

[38] Gogos AJ Young JS Morshed RA Avalos LN Noss RS Villanueva-Meyer JE. Triple motor mapping: transcranial, bipolar, and monopolar mapping for supratentorial glioma resection adjacent to motor pathways. J Neurosurg (2020) 134(6):1728–37. 10.3171/2020.3.JNS193434 32502996

[39] Moiyadi A Velayutham P Shetty P Seidel K Janu A Madhugiri V. Combined motor evoked potential monitoring and subcortical dynamic mapping in motor eloquent tumors allows safer and extended resections. World Neurosurg (2018) 120:e259–68. doi: 10.1016/j.wneu.2018.08.046 30138733

[40] Neuloh G Schramm J. Are there false-negative results of motor evoked potential monitoring in brain surgery? Cent Eur Neurosurg (2009) 70(4):171–5. doi: 10.1055/s-0029-1225651 19851956

[41] Senft C Forster MT Bink A Mittelbronn M Franz K Seifert V. Optimizing the extent of resection in eloquently located gliomas by combining intraoperative MRI guidance with intraoperative neurophysiological monitoring. J Neurooncol (2012) 109(1):81–90. doi: 10.1007/s11060-012-0864-x 22528791

